# Draft Genome Sequence of *Cryptococcus terricola* JCM 24523, an Oleaginous Yeast Capable of Expressing Exogenous DNA

**DOI:** 10.1128/genomeA.01238-16

**Published:** 2016-11-03

**Authors:** Dan Close, John Ojumu, Gui Zhang

**Affiliations:** a Biosciences Division, Oak Ridge National Laboratory, Oak Ridge, Tennessee, USA; b Coulter Department of Biomedical Engineering, Georgia Institute of Technology, Atlanta, Georgia, USA

## Abstract

*Cryptococcus terricola* JCM 24523 has recently been identified as an oleaginous yeast capable of converting starch into fatty acids. This draft genome sequence provides a platform for elucidating its fatty acid production potential and supporting comparisons with other oleaginous species.

## GENOME ANNOUNCEMENT

*Cryptococcus terricola* is an oleaginous yeast species from the basidiomycete family that is frequently found in soil samples (1, 2). Recently, strain JCM 24523 was discovered to be highly efficient at converting soluble starch-based waste products into fatty acids, and it has been suggested as a candidate for the consolidated bioprocessing of hydrocarbon chemicals (3). An analysis of this strain’s lipid production profile when grown on starch revealed that it favored the synthesis of unsaturated 18-carbon fatty acids (3), while growth in glucose-containing rich medium elicited an increase in the production levels of saturated 16- and 18-carbon fatty acids as well. Promisingly, work by our group has demonstrated that this strain is capable of expressing exogenous plasmid DNA. This may permit genetic modifications aimed at altering or improving its fatty acid synthesis production potential.

Although the natural fatty acid products of *C. terricola* JCM 24523 are amenable for use as biofuels or other high-value products (4), its ability to grow using low-cost starch feedstocks (3) and potential for genetic modification suggest it may be a candidate for further development as an industrial fermentation host. This draft genome sequence represents a first step toward elucidating the genetic mechanisms governing the enactment of its oleaginous metabolism and will aid in the identification of genomic targets for modification.

To obtain a sufficient quantity of DNA for sequencing, and because growth on glucose or starch did not yield significantly different fatty acid production profiles, *C. terricola* JCM 24523 was grown in YPD broth (10 g/liter yeast extract, 20 g/liter peptone, 20 g/liter glucose) at 25°C and 150 rpm. Under these conditions, metabolism favored cell growth over fatty acid production for increased DNA synthesis. DNA was collected from cells during mid-log-growth phase by lysing with 8 M urea, 0.5 M NaCl, 20 mM Tris, 20 mM EDTA, and 2% SDS and was isolated using a phenol-chloroform–isoamyl alcohol extraction, as previously described (5). The isolated DNA was treated with RNase to remove any contaminating RNA, and the residual RNase and digested RNA were removed by ethanol precipitation.

To prepare for sequencing on an Illumina HiSeq 2500 platform, both shotgun and mate-pair libraries were generated. The shotgun library insert size was determined to be approximately 324 bp and produced 7,208,260 sequence reads. The mate-pair library was determined to be approximately 6.5 kb and produced 12,040,344 sequence reads. The AllPaths LG whole-genome shotgun assembler (release version R49403) (6) was used to create a *de novo* genome assembly. A total of 1,178 contigs were produced, which were assembled into 156 scaffolds at an average read length of 148.6 kb. The maximum scaffold length was 1.75 Mb, and the scaffold *N*_50_ was 13 kb. This assembly represented an 84.3× coverage of the genome. The total genome size was determined to be 23.19 Mbp, with an overall G+C content of 50.9%.

### Accession number(s).

This whole-genome shotgun project has been deposited in DDBJ/ENA/GenBank under accession no. MATT00000000. The version described in this paper is the first version, MATT01000000.
